# Acyl-Carnitines Exert Positive Effects on Mitochondrial Activity under Oxidative Stress in Mouse Oocytes: A Potential Mechanism Underlying Carnitine Efficacy on PCOS

**DOI:** 10.3390/biomedicines11092474

**Published:** 2023-09-06

**Authors:** Martina Placidi, Teresa Vergara, Giovanni Casoli, Irene Flati, Daria Capece, Paolo Giovanni Artini, Ashraf Virmani, Samuele Zanatta, Anna Maria D’Alessandro, Carla Tatone, Giovanna Di Emidio

**Affiliations:** 1 Department of Life, Health and Experimental Sciences, University of L’Aquila, 67100 L’Aquila, Italy; martina.placidi@univaq.it (M.P.); teresa.vergara@guest.univaq.it (T.V.); giovanni.casoli@student.univaq.it (G.C.); annamaria.dalessandro@univaq.it (A.M.D.); carla.tatone@univaq.it (C.T.); 2 Department of Biotechnological and Applied Clinical Sciences, University of L’Aquila, 67100 L’Aquila, Italy; irene.flati@graduate.univaq.it (I.F.); daria.capece@univaq.it (D.C.); 3 Department of Obstetrics and Gynecology “P. Fioretti”, University of Pisa, 56126 Pisa, Italy; pg.artini@gmail.com; 4 Research, Innovation and Development, Alfasigma B.V., 3528 BG Utrecht, The Netherlands; ashraf.virmani@alfasigma.com; 5 Research and Development, Labomar Spa, 31036 Istrana, Italy; samuele.zanatta@labomar.com

**Keywords:** oocyte, mitochondria, propionyl-l-carnitine (PLC), l-carnitine (LC), acetyl-l-carnitine (ALC), carnitine palmitoyltransferase-1 (CPT1), oxygen consumption rate (OCR), fatty acid beta-oxidation, oxidative stress, polycystic ovarian syndrome (PCOS)

## Abstract

Carnitines play a key physiological role in oocyte metabolism and redox homeostasis. In clinical and animal studies, carnitine administration alleviated metabolic and reproductive dysfunction associated with polycystic ovarian syndrome (PCOS). Oxidative stress (OS) at systemic, intraovarian, and intrafollicular levels is one of the main factors involved in the pathogenesis of PCOS. We investigated the ability of different acyl-carnitines to act at the oocyte level by counteracting the effects of OS on carnitine shuttle system and mitochondrial activity in mouse oocytes. Germinal vesicle (GV) oocytes were exposed to hydrogen peroxide and propionyl-l-carnitine (PLC) alone or in association with l-carnitine (LC) and acetyl-l-carnitine (ALC) under different conditions. Expression of carnitine palmitoyltransferase-1 (Cpt1) was monitored by RT-PCR. In in vitro matured oocytes, metaphase II (MII) apparatus was assessed by immunofluorescence. Oocyte mitochondrial respiration was evaluated by Seahorse Cell Mito Stress Test. We found that Cpt1a and Cpt1c isoforms increased under prooxidant conditions. PLC alone significantly improved meiosis completion and oocyte quality with a synergistic effect when combined with LC + ALC. Acyl-carnitines prevented Cpt1c increased expression, modifications of oocyte respiration, and ATP production observed upon OS. Specific effects of PLC on spare respiratory capacity were observed. Therefore, carnitine supplementation modulated the intramitochondrial transfer of fatty acids with positive effects on mitochondrial activity under OS. This knowledge contributes to defining molecular mechanism underlying carnitine efficacy on PCOS.

## 1. Introduction

Carnitine is a generic term for the endogenous carnitine pool, which is present in cells and tissue as free l-carnitine (trimethylamino- β-hydroxybutyrate, LC) or acyl-carnitines. The short-chain carnitine esters include acetyl-l-carnitine (ALC) and propionyl-l-carnitine (PLC), characterized by different activity and pharmacokinetics properties [1]. Carnitines play a key physiological role in lipid metabolism and intermediary metabolic pathways [1,2,3]. Through the carnitine shuttle, LC helps in transporting the long-chain fatty acids from the cytoplasm to the mitochondrial matrix for subsequent degradation for beta-oxidation. By shuttling acetyl groups from outside to inside the mitochondrial membrane, carnitines regulate energetic metabolism and sense cellular energy level [1]. For proper cell functioning, the whole carnitine pool needs to be present, with LC representing 50–85% of the pool and ALC being the most abundant form of acyl-carnitines. Given the different effects of LC and ALC on cellular respiration, the combined presence of LC and ALC has been known to support metabolic flexibility [4]. The ALC/LC ratio ranging from 0.3 to 0.5 is tissue-specific and its changes are evidence of disturbed mitochondrial metabolism [1]. Fatty acid oxidation represents an important energy source for the oocyte, as evidenced by the observation that it is increased by LC exposure of cumulus–oocyte complexes during in vitro maturation and in vitro follicle culture in association with improved oocyte competence [5,6]. L-carnitine uses the Na^+^-driven LC/organic cation transporter-2 (OCTN-2) for its transport into the oocytes, where it is converted to ALC by carnitine palmitoyltransferase-1 (CPT1) in the outer mitochondrial membrane. For this reason, CPT1 is considered the rate-limiting enzyme of fatty acid metabolism [7]. To maintain low levels of reactive oxygen species (ROS) production, the mammalian oocyte requires a balance of pyruvate and fatty acid oxidation. The potential of carnitines to regulate lipid utilization with positive effects on energetic metabolism and oocyte competence has been very poorly investigated [8].

Carnitine administration seems to alleviate some symptoms of polycystic ovarian syndrome (PCOS), a metabolic and endocrine condition affecting 4–21% of women in reproductive age [9,10,11]. This disorder is associated with metabolic syndrome, obesity, or other comorbidities [12,13]. In recent years, there has been an increase in the prevalence of PCOS, which can lead to endometrial dysfunction, infertility, cardiovascular disease, and obesity, as well as insulin resistance, hyperinsulinemia, and hyperandrogenemia [14]. It has been reported that carnitines improve ovulation, clinical pregnancy, insulin resistance, and body mass index (BMI) in infertile women with PCOS along with having positive effects on serum lipid profile. Nevertheless, low to moderate certainty of evidence about these effects is reported [15,16,17], thereby stimulating further investigation.

A possible rationale for beneficial effects of carnitines on PCOS is that serum concentration of total carnitines was reduced in PCOS patients [12,18]. However, when PCOS patients were compared with controls with the same BMI, it emerged that carnitine deficiency was linked to the obese phenotype, a condition known to be associated with carnitine alterations [18] at systemic but also at intrafollicular levels [19]. Interestingly, a metabolomic study reported that levels of some carnitines are altered in the follicular fluid of PCOS women [20,21]. Consistently, administration of different carnitines in PCOS animal models resulted in beneficial effects on the ovarian microenvironment, as related to reduced oxidative damage, and improved mitochondrial activity, as well as in the recovery of oocyte competence in terms of spindle configuration [8,22].

It is acknowledged that carnitines have antioxidant properties by acting as scavengers of ROS, modulating the activities of ROS-producing enzymes and protecting mitochondrial metabolism [23,24]. Nevertheless, the hypothesis that carnitines may act directly at the oocyte level by alleviating the effects of a redox imbalance remains to be investigated. Oxidative stress (OS) is one of the main factors involved in the pathogenesis of PCOS [25,26,27,28,29,30,31,32]. In the context of OS, cellular and biochemical dysfunctions may be involved. The most relevant example is the relationship between PCOS and mitochondrial dysfunction [33,34,35]. It has been suggested that decreased mitochondrial oxygen consumption, glutathione, and increased ROS contribute to mitochondrial dysfunction in PCOS patients [34]. Follicular fluid in women with PCOS demonstrated increased levels of ROS and molecular oxidative damage. Total antioxidant capacity decreased in association with reduced oocyte maturation and fertilization rates, poor embryo quality, and lower pregnancy rates [36,37,38,39].

In the present study, we hypothesized that beneficial effects of carnitines administration previously observed on oocytes from a PCOS mouse model [8] may be related to the carnitine action against OS as the most known molecular alteration in the PCOS follicle microenvironment. We investigated the ability of different acyl-carnitines to act at the oocyte level by counteracting the effects of OS on carnitine shuttle system and mitochondrial activity in mouse oocytes. ALC and LC were tested in a ratio of 0.5 according to [8], at concentrations used in previous in vitro studies on mammalian oocytes [40,41,42,43], whereas protective effects of PLC were investigated under conditions used in other in vitro systems [44,45]. 

## 2. Materials and Methods

### 2.1. Animals

Outbred CD-1 mice (Charles River Laboratories Italia s.r.l., Calco, Italy) at the age of 4–8 weeks were maintained in a temperature-controlled environment under a 12 h light/dark cycle (07:00 a.m.–7:00 p.m.) with free access to feed and water ad libitum. All the experiments were carried out in conformity with national and international laws and policies (European Economic Community Council Directive 86/609, OJ 358, 18 December 1986; Italian Legislative Decree 116/92, Gazzetta Ufficiale della Repubblica Italiana n. 40, 18 February 1992; National Institutes of Health Guide for the Care and Use of Laboratory Animals, NIH publication no. 85–23, 1985). The project was approved by the Italian Ministry of Health and the Internal Committee of the University of L’Aquila (authorization n. 329/2022-PR). Mice were sacrificed by an inhalant overdose of carbon dioxide (CO_2_, 10–30%), followed by cervical dislocation. All efforts were made to minimize suffering.

### 2.2. Oocyte Collection and Treatments

Ovarian immature oocytes at the germinal vesicle stage (GV) were harvested after puncture of the antral follicles. The meiotic block at GV stage was achieved by addition of 0.5 μM cilostamide (Sigma-Aldrich, St. Louis, MO, USA) [46,47] in the culture media, M2 or M16 (Sigma-Aldrich), according to each procedure.

To induce OS, GV-blocked oocytes were exposed to 100 µM H_2_O_2_ for 10 min, according to Di Emidio et al. [48]. GV oocytes were then extensively washed and processed for further analysis. 

After the induction of OS, the oocytes were cultured for 16 h (in vitro maturation, IVM) in M16 supplemented with different concentrations and compositions: a mixture of LC (C0158, Sigma-Aldrich) and ALC (A6706, Sigma-Aldrich), at a 0.5 ratio of ALC/LC at the concentrations of 0.04, 0.4, and 1.6 mg/mL (equivalent to 0.25, 2.5, and 9.9 mM, respectively) LC and 0.02, 0.2, and 0.8 mg/mL (equivalent to 0.08, 0.8, and 3.3 mM, respectively) ALC; or PLC (42602, Sigma-Aldrich) alone at concentration of 0.2, 1.0, and 2.0 mg/mL (1, 5, and 10 mM, respectively); or a mixture of all tested carnitines LC, ALC, and PLC at the concentration 0.4, 0.2, and 0.2 mg/mL (equivalent to 2.5, 0.8, and 1.0 mM), respectively, representing the minimum effective doses of LC–ALC and PLC. At the end of IVM, numbers of oocytes that emitted first polar body (MII, metaphase II stage), oocytes that had resumed meiosis (GVBD, germinal vesicle breakdown stage), immature oocytes, and degenerated oocytes were recorded. MII oocytes were processed for the immunofluorescence analysis of the meiotic spindle. 

In order to test the beneficial effects of carnitine supplementation on OS damage, GV-blocked oocytes were incubated in M16 medium supplemented with PLC 0.2 mg/mL or the mixture of LC, ALC, and PLC (0.4, 0.2, and 0.2 mg/mL, respectively) for 2 h at 37 °C, 5% CO_2_, prior to exposure to 100 μm H_2_O_2_ for 10 min. GV oocytes were then processed for further analysis.

### 2.3. Analysis of DNA Distribution and Spindle Configuration of In Vitro Matured MII Oocytes

Metaphase II stage oocytes were fixed for immunofluorescence and labeled by mouse anti α-tubulin (1:200, T9026, Sigma Aldrich,) primary antibody overnight at 4 °C and secondary goat anti mouse-antibody conjugated with DyLight^®^ 594 (1:500, A90-137D4, Bethyl Laboratories Inc., Montgomery, TX, USA) for 1 h at room temperature. Chromatin staining was performed by 5 μg/mL Hoechst 33342 (Sigma-Aldrich) for 5 min at room temperature. In negative-control oocytes, the primary antibody was omitted. Oocytes were mounted on slides and analyzed under epifluorescence microscope at 100× magnification. 

**Figure 1:**
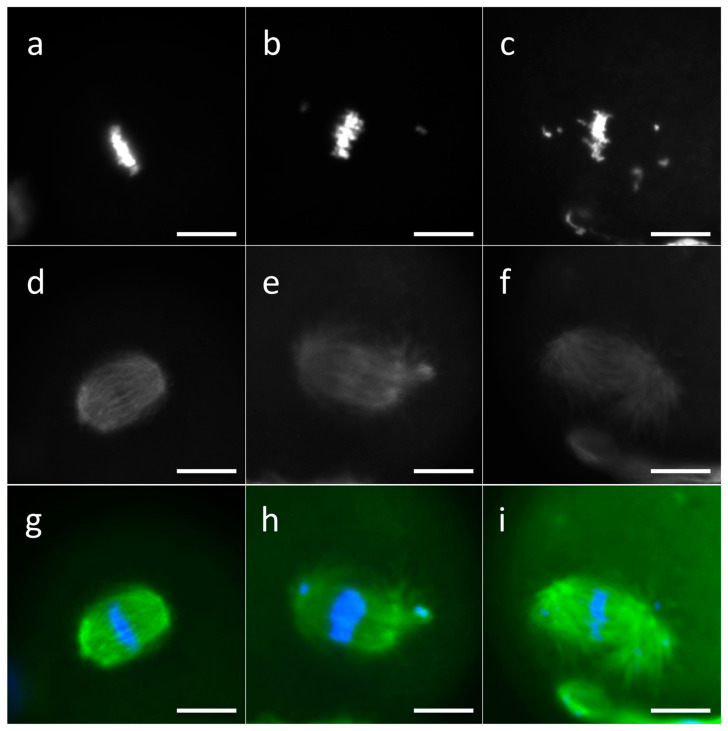
Representative images of MII plate in MII oocytes. Chromosomes were classified as normal (**a**), slightly aberrant (**b**), or aberrant (**c**). Spindle was classified as normal (**d**), slightly aberrant (**e**), or aberrant (**f**). Merged images of DNA and spindle staining are presented (**g**–**i**). MII oocytes were labeled by mouse anti α-tubulin primary antibody and secondary antibody conjugated with DyLight^®^ 594, and chromatin staining was performed by Hoechst 33342. Scale bars: 10 μm.

Based on the characteristics of the meiotic spindle, the oocytes were classified as (i) normal, characterized by a correctly assembled and bipolar mitotic spindle and correct chromosome alignment; (ii) slightly aberrant, characterized by a meiotic spindle with slight disorganization of the microtubules or with a slightly abnormal structure and/or a slight dispersion of the chromosomes, with up to four scattered chromosomes; (iii) aberrant, characterized by a completely disorganized or abnormal or absent mitotic spindle and/or a total DNA disorganization on the MII plate or by the presence of noncondensed chromosomes (Figure 1). 

### 2.4. RNA Extraction and Real-Time Reverse Transcriptase-Polymerase Chain Reaction Analysis 

Total RNA was extracted from oocytes by using a PicoPure™ RNA Isolation Kit (KIT0204, Applied Biosystems, Foster City, CA, USA). According to the protocol of the reverse transcription kit (NP100042, OriGene Technologies, Rockville, MD, USA), RNA (1 μg) was converted into complementary DNA (cDNA). The cDNA was used for reverse transcriptase-polymerase chain reaction (RT-PCR) reactions, using the Applied Biosystems 7300 system (Thermo Fisher Scientific, Inc., Rockford, IL, USA), the TaqMan^®^ Gene Expression Master Mix (4444557, Applied Biosystems) and TaqMan gene expression assays FAM-MGB (Applied Biosystems), in accordance with the supplier’s instructions. The assays used were 18S-Mm03928990_g1, CPT1a- Mm01231183_m1, and CPT1c- Mm01202530_g1. Amplification steps were set as follows: 2 min at 50 °C and 20 s at 95 °C for the initial maintenance phase, followed by 40 cycles for 1 s at 95 °C and 20 s at 60 °C. Gene expression was calculated using the ΔΔCt method [49], by normalization of the level of each transcript to that of 18S and to the normalized level of transcript of the control group.

### 2.5. Application of Seahorse XFp to Measure Oxygen Consumption in Oocytes 

Sensors containing Seahorse flux packs (Agilent Technologies, Santa Clara, CA, USA) were incubated overnight at 37 °C in a non-CO_2_ humidified incubator and calibrated according to manufacturer’s instructions. Oocytes were analyzed using a specialized protocol involving a 15 min equilibration period upon loading the cell plate and alternating between a 3 min measurement period and a 1 min re-equilibration period. The measurement period involved the lowering of a sensor-containing probe, which created an airtight 2.3 μL microenvironment in which change in pressure in mmHg was measured over time. This was followed by a 1 min period in which the probe was lifted, and the 180 μL well re-equilibrated. Plate specific ‘blank’ cell-free wells containing culture medium were used to account for environmental changes and flux of oxygen in the absence of cells. Mitochondrial inhibitors were dissolved in 100% DMSO or medium and diluted in warmed analysis media within 30 min from the beginning of the assay. Optimization was performed in GV oocytes to establish appropriate concentrations of mitochondrial inhibitors (oligomycin, carbonyl cyanide-p-trifluoromethoxyphenyl-hydrazone, FCCP, and a combination of antimycin A and rotenone, A/R, was obtained as per Seahorse XF Cell Mito Stress Test Kit, 103015-100, Agilent Technologies; 2,4-dinitrophenol, 2,4-DNP, D198501, Sigma-Aldrich) [50,51,52]. 

We tested oligomycin in concentrations ranging from 0.5 to 2 μM. The establishment of the most suitable uncoupler was based on the comparison between the effects of FCCP, as per the Seahorse XF Cell Mito Stress Test Kit instructions, and 2,4-DNP, using concentrations ranging from 1 to 7.5 μM and 100 to 250 μM, respectively, with 2,4-DNP being identified as the most effective. Antimycin A/rotenone were tested at concentrations from 1 to 3 μM. 

Mitochondrial bioenergetic profile was established after serial injections of (1) oligomycin, (2) 2,4-DNP, and (3) A/R. By blocking ATP synthase, oligomycin allows the measurement of ATP-linked respiration. The addition of mitochondrial uncoupler is used to collapse the inner mitochondrial membrane gradient in order to maximize the electron transport chain (ETC) functioning capacity. Finally, combined addition of rotenone and antimycin A blocks the ETC and reveals the residual, nonmitochondrial respiration [52]. Moreover, other functions can be extrapolated from the analysis of oxygen consumption rate (OCR) in response to mitochondrial inhibitors. In particular, proton leak can be obtained by subtracting nonmitochondrial respiration from the value of ATP-linked respiration, and spare respiratory capacity (SRC), by subtracting basal respiration from the value of maximal respiration. This allowed us to analyze and compare total respiration (baseline OCR prior to inhibitor injection), nonmitochondrial respiration, proton leak, SRC, and ATP production in the different experimental groups.

The analysis was performed on pools of 6–8 oocytes. Each measurement was performed at least in triplicate. Wave software (Agilent Technologies, Santa Clara, CA, USA) was used to determine oxygen consumption in pmol/min/well. This value was normalized to number of oocytes per well. Comparisons were performed only on data obtained within the same measurement session. 

### 2.6. Analysis of ATP Production

Measurements of cytosolic ATP levels were performed according to Cell Titer-Glo ATP assay kit (Promega Ltd., Southampton, UK). Briefly, pools of 3–7 oocytes were placed in M2 medium in 96-well white bottom plates (Corning, New York, NY, USA). The light signals were taken as the steady-state values. Light was recorded using a VIKTOR3^TM^ luminometer (PerkinElmer, Waltham, MA, USA). The signals were calibrated with a series of dilutions of ATP (A26209, Sigma-Aldrich) ranging from 0–50 nM.

### 2.7. Statistical Analysis

Analyses were performed using Sigma Stat software (Jandel Scientific Corporation, Leighton Buzzard, UK). All data are presented as mean ± standard error of the mean (SEM). Statistical analysis was carried out using Student’s t-test. A *p*-value of <0.05 was considered statistically significant.

## 3. Results

### 3.1. Analysis of Cpt1a and Cpt1c Expression in Oocytes Exposed to Oxidative Stress

**Figure 2:**
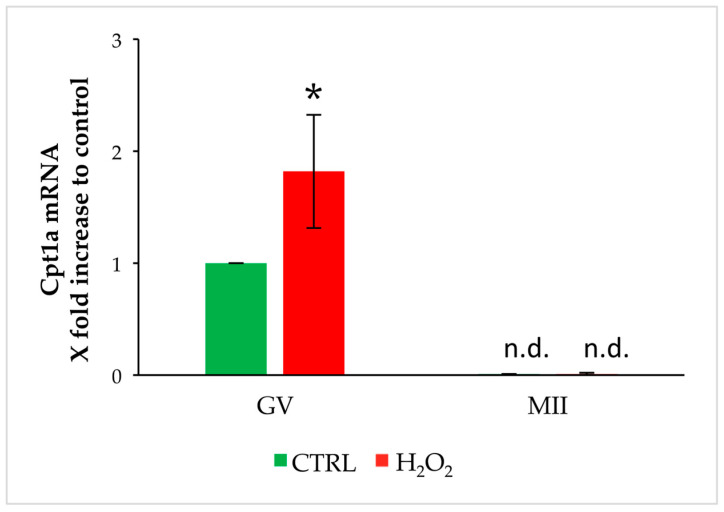
Changes in gene expression of Cpt1a in GV and MII oocytes stressed by H_2_O_2_. Values are means ± SEM of three determinations. The fold change of each gene is shown as 2^−ΔΔCt^. Statistical comparison was performed by *t*-test. * *p* < 0.05 vs. control; n.d.: not detectable.

In order to investigate the role of OS exposure on changes of oocyte metabolism, we analyzed the transcript levels of Cpt1a and Cpt1c. To this aim, we exposed GV oocytes to OS and evaluated the transcript level soon after exposure or after 16 h culture for IVM in oocytes reaching the MII stage. As shown in Figure 2, Cpt1a is expressed in GV oocytes but is undetectable after IVM. Exposure to OS increases its expression in GV oocytes in comparison to control, but its levels decline to undetectable levels after IVM. 

**Figure 3:**
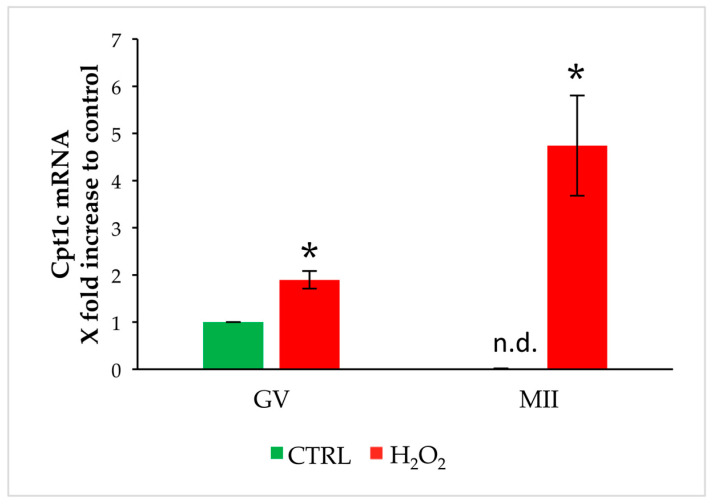
Changes in gene expression of Cpt1c in GV and MII oocytes stressed by H_2_O_2_. Values are means ± SEM of three determinations. The fold change of each gene is shown as 2^−ΔΔCt^. Statistical comparison was performed by *t*-test. * *p* < 0.05 vs. control; n.d.: not detectable.

As described for Cpt1a, Cpt1c is expressed in GV oocytes but is undetectable after IVM. Exposure to OS increases its expression in GV oocytes in comparison to control, and meiotic resumption leads to an increase in Cpt1c levels (Figure 3).

### 3.2. Effects of L-Carnitine and Acetyl-L-Carnitine on In Vitro Maturation and MII Configuration of Oocytes Exposed to Oxidative Stress

Based on changes induced by exposure to OS on Cpt1a and Cpt1c gene expression, we tested the effects of medium supplementation with a mixture of LC and ALC. As expected, exposure to OS reduced the percentage of oocytes that completed meiosis after 16 h of IVM and emitted the first polar body, when compared to the control (Table 1). The presence of 0.2 mg/mL ALC and 0.4 mg/mL LC in the IVM medium was the minimum effective dose in counteracting the negative detrimental effects of H_2_O_2_ on meiosis resumption and polar body extrusion, reaching levels similar to the control group. Lower concentrations of ALC and LC were unable to counteract the effects induced by H_2_O_2_.

As shown in Table 2, exposure to oxidative treatment reduced the percentage of oocytes that showed a normal metaphase plate and increased the number of oocytes with aberrant configuration compared to the control. The addition of ALC and LC to the culture medium after exposure to OS did not improve the percentage of oocytes showing normal spindle and chromosome configuration after IVM in comparison to oocytes matured in simple medium after exposure to H_2_O_2_. Interestingly, the addition of ALC and LC during IVM after exposure to H_2_O_2_ increased the rate of oocytes with aberrant MII configuration when compared to the H_2_O_2_ group. In comparison to the control, the addition of ALC and LC did not counteract the detrimental effects on MII spindle and chromosomes, and the highest dose (0.8 mg/mL ALC + 1.6 mg/mL LC) decreased the percentage of normal oocytes.

Therefore, although medium supplementation with ALC–LC in oocytes subjected to IVM after oxidative insult showed beneficial effects on maturation rate, oxidative damage on MII spindle and chromosome configuration was not prevented.

### 3.3. Effects of Propionyl-L-Carnitine on In Vitro Maturation and MII Configuration of Oocytes Exposed to Oxidative Stress

Recently, the beneficial role of PLC in modulating energy metabolism as strategy to improve oocyte quality and female reproductive performance has emerged [8,53,54]. Therefore, since ALC and LC were not effective in preventing oxidative damage in MII oocytes, we tested whether exposure to PLC could produce beneficial effects both on meiotic resumption and MII plate perturbations. As shown in Table 1, PLC counteracted oxidative effects on polar body emission after 16 h of IVM at all tested concentrations: 0.2, 1.0, and 2.0 mg/mL. All concentrations were able to restore IVM rate to levels similar to control oocytes.

According to Table 2, after exposure to OS, the addition of 0.2, 1.0, or 2.0 mg/mL PLC to the culture media was able to significantly increase the number of oocytes with a normal MII spindle and chromosome configuration in comparison to H_2_O_2_, reaching the same levels as the control. Nevertheless, the percentage of oocytes with slightly aberrant and aberrant MII plate was similar to H_2_O_2_.

### 3.4. Effects of Medium Supplementation with a Mixture of L-Carnitine, Acetyl-L-Carnitine, and Propionyl-L-Carnitine on In Vitro Maturation and MII Configuration of Oocytes Exposed to Oxidative Stress

Based on previous results, we tested potential synergistic effects of LC, ALC, and PLC on oocytes’ competence for meiosis resumption following OS. To this end, medium was supplemented with minimum effective doses of LC–ALC and PLC. As shown in Table 1, the presence of 0.2 mg/mL PLC, 0.2 mg/mL ALC, and 0.4 mg/mL LC in the IVM medium prevented the effects of H_2_O_2_ on first polar body emission by maintaining the same percentage of MII oocytes observed in the control group. 

Regarding MII plate, the addition of the mixture of carnitines had beneficial effects on preventing oxidative damage. In particular, in comparison to oocytes exposed to H_2_O_2_, the addition of LC–ALC–PLC increased the number of oocytes showing normal MII spindle and chromosome configuration and decreased the percentages of oocytes showing slightly aberrant and aberrant MII plate. The aberration pattern of MII plate in oocytes exposed to the mixture of carnitines after oxidative insult was similar to the control group.

### 3.5. Effects of Carnitines on Cpt1a and Cpt1c Gene Expression in Oocytes Exposed to Oxidative Stress

**Figure 4:**
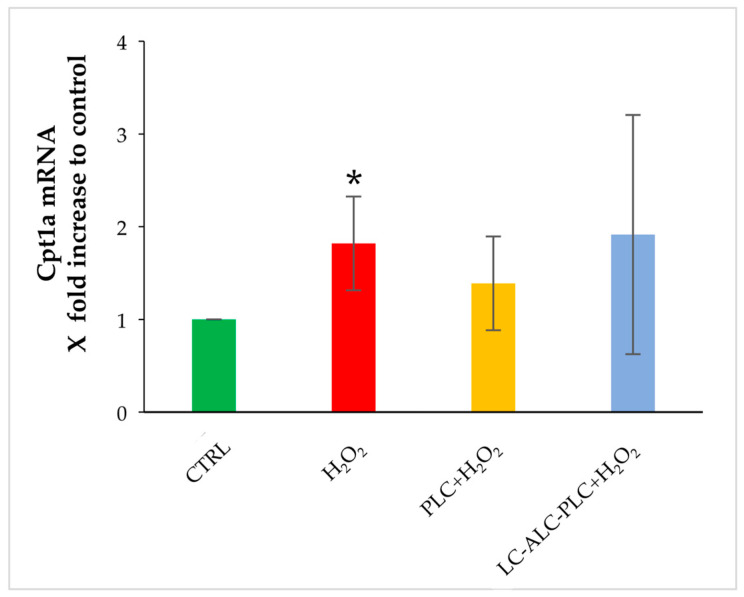
Changes in gene expression of Cpt1a in GV oocytes exposed to PLC or to LC–ALC–PLC for 2 h prior to stress by H_2_O_2_. Values are means ± SEM of three determinations. The fold change of each gene is shown as 2^−ΔΔCt^. Statistical comparison was performed by *t*-test. * *p* < 0.05 vs. control.

In the second part of the study, we compared the effect of the exposure of GV oocytes to PLC or to LC–ALC–PLC for 2 h prior to H_2_O_2_ on Cpt1a and Cpt1c gene expression. The oxidative insults induced the increase in Cpt1a in comparison to control oocytes. The exposure to PLC or the mixture of LC–ALC–PLC was not able to prevent this increase (Figure 4).

**Figure 5:**
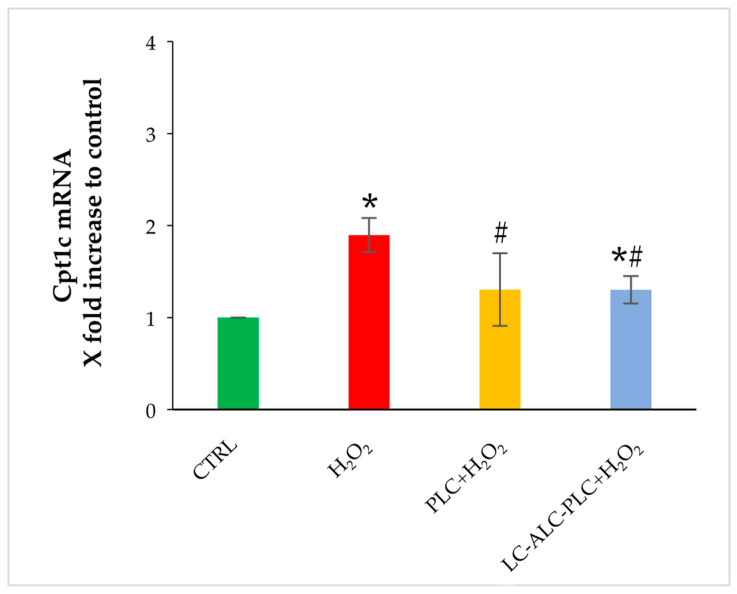
Changes in gene expression of Cpt1c in GV oocytes exposed to PLC or to LC–ALC–PLC for 2 h prior to H_2_O_2_. Values are means ± SEM of three determinations. The fold change of each gene is shown as 2^−ΔΔCt^. Statistical comparison was performed by *t*-test. * *p* < 0.05 with respect to the control; ^#^ *p* < 0.05 compared to H_2_O_2_.

As observed for Ctp1a, the exposure to H_2_O_2_ induced the transcript expression of Cpt1c. Preincubation with PLC prevented the Cpt1c increase induced by the oxidative insult and restored its expression to levels similar to control. Incubation with the mixture of LC–ALC–PLC prior to OS exposure lowered the expression of Cpt1c in comparison to the H_2_O_2_ group. In comparison to control, the mixture was not able to restore Cpt1c expression to the basal level (Figure 5). 

### 3.6. Establishment of Mitochondrial Inhibitors for Measurements of Oxygen Consumption Rate 

Before proceeding with the measurements of oxygen consumption, we had to perform a titration of each mitochondrial drug in order to establish the minimum effective dose (MED). 

**Figure 6:**
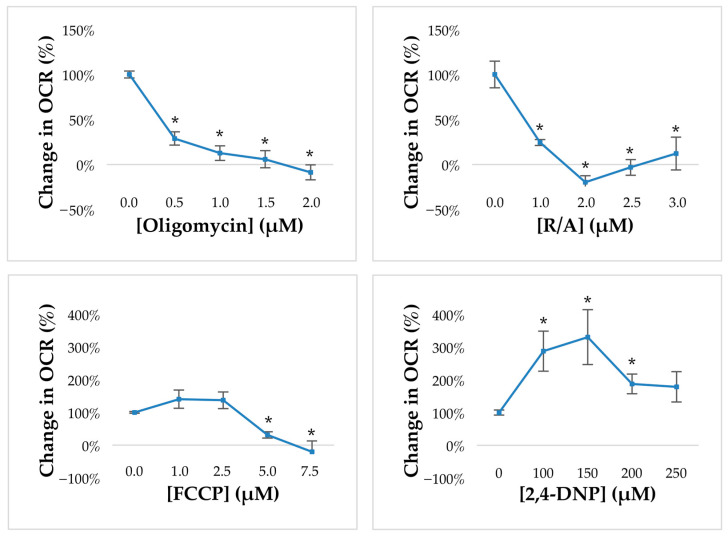
Optimization of drug concentrations used for dissecting the components of oxygen consumption in mouse GV oocytes. Data show mean ± SEM. Statistical comparison was performed by *t*-test. * *p* < 0.05.

The first drug to be tested was oligomycin, which inhibits ATP synthase and can be used to indicate the proportion of O_2_ consumption directly coupled to ATP generation. In order to optimize oligomycin dose, we tested concentrations ranging from 0.5 to 2 μM. As shown in Figure 6, 0.5 µM oligomycin induced a 75% reduction of OCR in mouse GV oocytes and was considered as the MED to be employed in the following experiments.

The next step was the selection of the most effective protonophore between FCCP and 2,4-DNP. Both drugs, also known as mitochondrial uncouplers, are used to collapse the inner membrane gradient, thus allowing the ETC to function at maximal capacity. Results on FCCP revealed that in mouse GV oocytes, FCCP did not induce a significant increase in OCR, while higher doses were associated with a toxic effect on cell respiration and survival. By contrast, 2,4-DNP, which has been largely employed in previous studies by our research group, doubled the OCR at the first dose (100 µM), which was maintained at the second dose prior to the toxic effect linked to massive proton uncoupling. Therefore, 100 µM 2,4-DNP was established as the mitochondrial uncoupler to be employed in the following experiments.

Finally, we tested a combination of antimycin A, a complex III inhibitor, and rotenone, a complex I inhibitor, in order to inhibit the ETC and unveil the residual, nonmitochondrial respiration. As shown in Figure 6, 1 μM A/R gave the maximal response by inducing a drop of more than 90% in OCR and was selected as MED for the following analysis.

### 3.7. Effect of PLC or Mixture of L-Carnitine, Acetyl-L-Carnitine, and Propionyl-L-Carnitine on Mitochondrial Activity of GV Oocytes Exposed to Oxidative Stress 

The exposure to OS increased the total basal respiration and oxygen consumption related to nonmitochondrial reactions in comparison to control oocytes. No changes were observed in proton leak. The spare respiratory capacity, indicating the capacity of cell to adapt to stressing conditions, was reduced after oxidative insult in comparison to control. Finally, in order to counteract oxidative damage, oocytes increased ATP production.

The exposure of GV oocytes to PLC (0.2 mg/mL) for 2 h prior to the treatment with H_2_O_2_ prevented the increased of total basal respiration and nonmitochondrial respiration induced by OS. Interestingly, PLC improved SRC in comparison with the H_2_O_2_ group, with levels higher than the control group. Similarly to control, ATP production was lower than the H_2_O_2_ group. 

**Figure 7:**
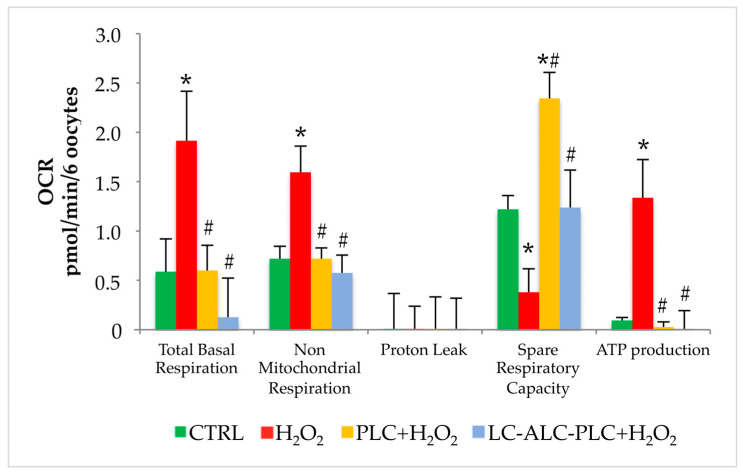
Comparison of bioenergetics profile of GV oocytes exposed to plain medium (CTRL), H_2_O_2_, or incubated with PLC or LC–ALC–PLC prior to H_2_O_2_ analyzed by extracellular flux analysis. Data show mean ± SEM. Statistical comparison was performed by *t*-test. * *p* < 0.05 vs. CTRL; # *p* < 0.5 vs. H_2_O_2_.

The addition of the mixture of LC, ALC, and PLC in medium of GV oocytes prior to OS maintained total basal respiration and nonmitochondrial respiration levels similar to control, preventing the increase induced by OS. The mixture improved the SRC with levels similar to control. Regarding ATP production, as observed in the PLC group, the mixture prevented the burst in energy production induced by oxidative insult (Figure 7).

### 3.8. Analysis of ATP Production

**Figure 8:**
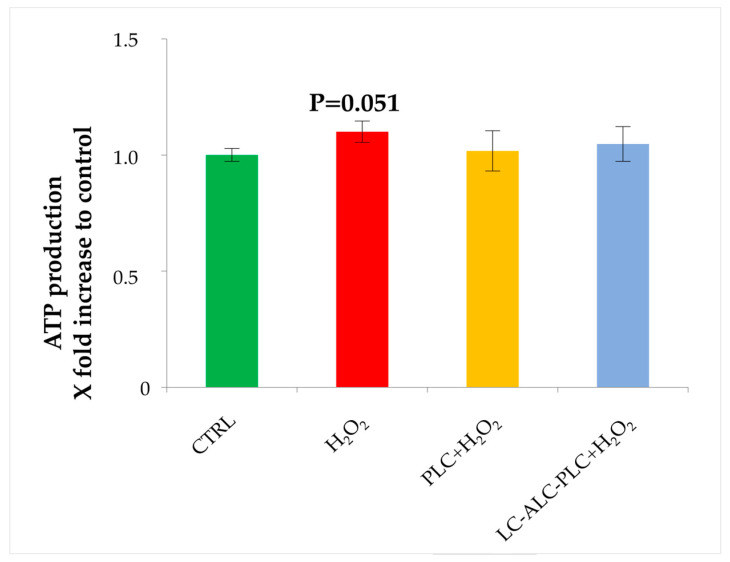
Analysis of ATP production of GV oocytes exposed to PLC or to LC–ALC–PLC for 2 h prior to H_2_O_2_. Data show mean ± SEM.

Unlike results obtained with OCR measurement, analysis of the ATP production did not reveal significant differences among groups. Nevertheless, in accordance with previous analysis, we obtained a trend of increased ATP production in the GV oocytes exposed to H_2_O_2_ in comparison to the control group (Figure 8).

## 4. Discussion

PCOS is a reproductive disease characterized by perturbations in serum carnitine levels, which are likely to be associated with obesity and insulin resistance. Since acyl-carnitines are essential for fatty acid intramitochondria transport, and altered fatty acid beta-oxidation has been reported in PCOS women, it has been hypothesized that carnitine deficiency may impact energetic metabolism [55,56,57]. Alterations in the carnitine pool have been observed at intrafollicular level, in association with metabolic changes suggesting an important role of these energetic modulators in the reduced oocyte competence associated with this syndrome [58].

In the present study, we investigated the hypothesis that perturbations of the carnitine system at the oocyte level can occur as a consequence of OS. To this end, we explored the effects of mild pro-oxidant conditions on the carnitine shuttle system along with competence and mitochondrial activity of mouse oocytes by investigating acyl-carnitines’ ability to counteract these changes. 

We analyzed the expression of two isoforms of Cpt1, the rate-limiting enzyme for fatty acid beta-oxidation responsible for the transport of acyl-CoA to the mitochondria matrix through the carnitine system [59]. Diseases like metabolic disorders and various cancers are known to result from CPT1 deficiency or abnormal regulation [60]. From our experiments, Cpt1a is expressed in mouse oocytes at the GV stage and becomes undetectable at the MII stage. These findings are consistent with previous studies showing the expression of this isoform in mammalian oocytes from ovarian follicles [61,62,63]. In contrast to Cpt1a, Cpt1c is known to have low catalytic activity and is mainly localized in the endoplasmic reticulum (ER) rather than in mitochondria [64,65,66]. CPT1C has been described almost exclusively in neurons, stem cells, and cancer cells [66,67,68]. Recently, transcriptomic studies in humans revealed Cpt1c expression in oocytes and granulosa cells [62,63]. To our knowledge, this study represents the first evidence showing that Cpt1c, similarly to Cpt1a, is expressed in mouse oocytes at the GV stage and becomes undetectable when the MII stage is reached upon IVM, suggesting the relevance of oocyte fatty acid metabolism during folliculogenesis rather than at fertilization. Interestingly, both the isoforms investigated in this study were found to increase in GV oocytes in response to mild pro-oxidant conditions. This supports the hypothesis that, in response to OS, oocytes activate the molecular machinery responsible for the carnitine-dependent transport of fatty acids into the mitochondria, with a probable increase in fatty acid beta-oxidation. A similar behavior was reported in somatic cells, where increased expression of CPT1A after oxidative insult was documented as supporting evidence of adaptation to OS [69,70]. When oocytes exposed to OS reached the MII stage, in contrast to Cpt1a, which remains undetectable, Cpt1c was found to greatly increase, indicating that this isoform plays a peculiar role in the oocyte antioxidant response. 

Recently, our research group demonstrated that different acyl-carnitine formulations modulate differently ovarian mitochondrial activity in a PCOS mouse model [8]. Considering that both the Cpt1 isoforms are upregulated upon OS, it can be speculated that oocytes may require carnitines to cope with the oxidative insult. Therefore, we investigated the efficacy of different acyl-carnitines in counteracting the effects of a redox imbalance mimicking an aspect of the PCOS ovarian microenvironment [39].

Increasing doses of LC and ALC at a 0.5 ratio of ALC/LC were effective in preventing the negative effects of OS on IVM rate but did not counteract the negative action on the metaphase configuration. By contrast, oocyte exposure to PLC alone exerted beneficial effects in terms of both meiosis completion and quality of mature oocytes. According to the literature, the greater efficacy of PLC, compared to LC and ALC, could be linked to the synergy of its antioxidant action with its effects on energy and mitochondrial metabolism by replenishing tricarboxylic acid cycle (TCA) intermediates from propionyl-CoA, stabilizing membranes and reducing lipid peroxidation [53,71,72]. The combined presence of the three carnitine forms at low concentrations is more beneficial than the highest concentration of ALC–LC or PLC in terms of improved oocyte maturation and spindle integrity after exposure to OS. Thus it is very likely that, although an interconversion of carnitines inside the cell is possible, our results provide evidence for a synergistic effect of these carnitines, probably allowing the re-establishment of the carnitine pool. Indeed, the composition of the carnitine pool is tightly regulated and severely hampered by the OS [73]. 

However, neither PLC alone nor the mixture of LC, ALC, and PLC counteracted the Cpt1a increase in response to oxidative insult. By contrast, all the carnitine treatments were able to prevent the increase of Cpt1c expression observed upon OS, confirming the peculiar role of this isoform. Overall, these findings suggest that carnitine supplementation modulates the transfer of fatty acids into the mitochondria to support the OS-dependent increase of beta-oxidation. On the other hand, these observations support the need for further studies on the role of the CPT1 isoforms in the oocytes, considering that in some cellular systems CPT1C has been found to be expressed also on ER, indicating a role different to that of the canonical CPT1 enzymes [64,74]. Recently, CPT1C has been proposed as a sensor of cell nutritional status and a hallmark of lipid metabolic adaptation. In cancer cells, CPT1C expression is known to regulate lipid metabolic reprograming and cell adaptation to environmental stressors. In neurons, it regulates metabolism and function by interacting with proteins involved in the regulation of lipid metabolism, Golgi-mediated secretory transport, and the endolysosomal system [75].

In the last part of the study, the hypothesis that the protective effects of acyl-carnitines against OS were mediated by changes in mitochondrial activity was investigated. The rate of respiration, in terms of OCR, has been used for the last decade as a noninvasive and strong indicator of metabolic activity and mitochondrial function [52]. Despite the advent of high-throughput respirometry and the availability of specific mitochondrial inhibitors, it has been scarcely applied in oocytes [50,51,76]. In the present study, we employed the real-time OCR assay in mouse oocytes by focusing on the impact of oxidative insult. We found a significant increase of total basal respiration and nonmitochondrial respiration, the latter being a condition related to cytoplasmic oxidative processes [77], whereas no differences were observed in proton leak, indicating that the integrity of the inner mitochondrial membrane was not altered by the oxidative insult used in this study. From OCR analysis also emerged an increased ATP production upon OS associated with a reduced spare respiratory capacity, suggesting that oocyte ability to increase energetic metabolism was decreased. Thus, the stressed oocytes had likely reached the maximum increase in energy production to cope with oxidative insult. The exposure of oocytes to PLC alone or the mixture of LC, ALC, and PLC significantly prevented the modification of oocyte respiration, supporting the hypothesis that acyl-carnitines modulate mitochondrial activity in response to OS. In accordance with the findings on expression levels of the Cpt1 isoforms, acyl-carnitines act against OS by modulating energy production by fatty acid beta-oxidation. By increasing spare respiratory capacity independently of the oxidative insult, it is likely that PLC alone is more efficient than the carnitine mixture in maintaining mitochondrial activity upon OS. 

The results obtained open new hypotheses on the role of carnitines and beta-oxidation of fatty acids in the energy homeostasis of germ cells, already proposed in previous studies [78]. In particular, from this work emerges the need to clarify the role of the CPT1C isoform in oocytes and the mechanism of action of l-carnitine and its esters, by measuring various cellular parameters associated with OS, such as the production of ATP and ROS. 

The study of cellular and molecular aspects of PCOS at the intraovarian level is challenging due to the failure in selecting suitable models [79,80]. Therefore, the in vitro system employed in the present study to demonstrate that specific acyl-carnitines act under OS with valuable improvement of oocyte energetic metabolism is not a valid system for modeling the complex microenvironment of the PCOS ovary. However, by recapitulating in vitro only OS, as a single and specific aspect of PCOS, we demonstrated that a mechanism underlying the beneficial effects of carnitine administration is represented by acyl-carnitines’ ability to counteract oocyte alterations induced by intrafollicular OS. Thus, whether carnitines can counteract all oocyte alterations induced by the complex cellular interactions that occur in the PCOS ovary remains to be established. 

Overall, the present study contributes to the understanding of molecular mechanisms underlying carnitine efficacy on PCOS and unveils new applications of carnitine therapy in other ovarian dysfunctions related to redox imbalance such as those occurring during aging or endometriosis. Further research is required to clarify the role of acyl-carnitines and their deficiencies in the pathophysiology of PCOS in order to consider acyl-carnitines as promising pharmacological agents in the management of this endocrine disorder.

## Figures and Tables

**Table 1:** Percentage of oocytes showing the first polar body after IVM ^1^.

Experimental Group	n	Degeneration (Mean% ± SEM)	GV (Mean% ± SEM)	GVBD (Mean% ± SEM)	MII (Mean% ± SEM)
CONTROL	192	2.2% ± 1.3	12.0% ± 4.4	13.8% ± 4.2	72.0% ± 4.2
H_2_O_2_ 100 µM	145	1.2% ± 0.7	15.9% ± 7.7	44.1% ± 2.9 *	36.7% ± 7.8 *
H_2_O_2_ 100 µM + (ALC 0.04 mg/mL − LC 0.08 mg/mL)	46	0.0% ± 0.0	16.3% ± 8.7	26.7% ± 11.7 ^#^	56.9% ± 3.1
H_2_O_2_ 100 µM + (ALC 0.2 mg/mL − LC 0.4 mg/mL)	53	2.0% ± 2.0	9.4% ± 1.4	9.4% ± 1.36 ^#^	73.1% ± 12.8 ^#^
H_2_O_2_ 100 µM + (ALC 0.8 mg/mL − LC 1.6 mg/mL)	47	0.0% ± 0.0	12.8% ± 0.8	12.8% ± 0.8 ^#^	72.1% ± 3.9 ^#^
H_2_O_2_ 100 µM + PLC 0.2 mg/mL	89	0.0% ± 0.0	18.8% ± 4.0	12.5% ± 12.5 ^#^	69.3% ± 9.3 ^#^
H_2_O_2_ 100 µM + PLC 1.0 mg/mL	42	0.0% ± 0.0	4.8% ± 1.2	4.8% ± 4.0 ^#^	90.5% ± 5.2 ^#^
H_2_O_2_ 100 µM + PLC 2.0 mg/mL	48	4.2% ± 1.2	1.1% ± 1.1	33.3% ± 3.8	62.5% ± 6.1 ^#^
H_2_O_2_ 100 µM + (PLC 0.2 mg/mL − ALC 0.2 mg/mL − LC 0.4 mg/mL)	68	0.0% ± 0.0	10.8% ± 3.4	17.5% ± 6.4 ^#^	71.7% ± 9.7 ^#^

^1^ Statistical comparison was performed by *t*-test. * *p* < 0.05 with respect to the control; ^#^ *p* < 0.05 compared to H_2_O_2_.

**Table 2:** Percentage of oocytes with a normal, slightly aberrant, and aberrant metaphasic plate conformation.

Experimental Group	n	Normal (Mean% ± SEM)	Slightly Aberrant (Mean% ± SEM)	Aberrant (Mean% ± SEM)
CONTROL	66	55.7% ± 13.0	32.5% ± 11.5	11.8% ± 1.4
H_2_O_2_ 100 µM	52	14.8% ± 5.9 *	51.5%± 6.1	33.6% ± 2.0 *
H_2_O_2_ 100 µM + (ALC 0.04 mg/mL − LC 0.08 mg/mL)	26	23.5% ± 9.5	7.0% ± 7.0 ^#^	69.5% ± 2.5 ^#,^*
H_2_O_2_ 100 µM + (ALC 0.2 mg/mL − LC 0.4 mg/mL)	38	22.5% ± 2.5	23.3%± 3.3 ^#^	54.2% ± 0.8 ^#^*
H_2_O_2_ 100 µM + (ALC 0.8 mg/mL − LC 1.6 mg/mL)	33	9.4% ± 3.9 *	27.2% ± 0.6 ^#^	63.3% ± 3.3 ^#,^*
H_2_O_2_ 100 µM + PLC 0.2 mg/mL	38	32.7% ± 3.9 ^#^	51.7% ± 21.2	15.5% ± 18.0
H_2_O_2_ 100 µM + PLC 1.0 mg/mL	19	42.1% ± 7.6 ^#^	47.4% ± 1.0	10.5% ± 8.6
H_2_O_2_ 100 µM + PLC 2.0 mg/mL	21	49.0% ± 11.1^#^	18.2% ± 1.2 ^#^	40.5% ± 9.6 *
H_2_O_2_ 100 µM + (PLC 0.2 mg/mL − ALC 0.2 mg/mL − LC 0.4 mg/mL)	36	90.9% ± 9.1 ^#^	9.1% ± 9.1 ^#^	0.0% ± 0.0 ^#^

Statistical comparison by *t*-test. * *p* < 0.05 with respect to the control; ^#^ *p* < 0.05 compared to H_2_O_2_.

## Data Availability

Data supporting the findings of this study are available from the corresponding author upon reasonable request.
